# A Comparative Analysis of ProTaper Ultimate and Five Multifile Systems: Design, Metallurgy, and Mechanical Performance

**DOI:** 10.3390/ma18061260

**Published:** 2025-03-13

**Authors:** Jorge N. R. Martins, Emmanuel João Nogueira Leal Silva, Victor Talarico Leal Vieira, Rui Pereira da Costa, Abayomi O. Baruwa, Francisco Manuel Braz Fernandes, Marco Aurélio Versiani

**Affiliations:** 1 Faculdade de Medicina Dentária, Universidade de Lisboa, 1600-277 Lisboa, Portugal; 2 LIBPhys-FCT UID/FIS/04559/2013, 1600-277 Lisboa, Portugal; 3 Grupo de Investigação em Bioquimica e Biologia Oral (GIBBO), Unidade de Investigação em Ciências Orais e Biomédicas (UICOB), 1600-277 Lisboa, Portugal; 4 Centro de Estudos de Medicina Dentária Baseada na Evidência (CEMDBE), 1600-277 Lisboa, Portugal; 5 Department of Endodontics, School of Dentistry, Grande Rio University (UNIGRANRIO), Rio de Janeiro 21210-623, Brazil; 6 Department of Endodontics, Fluminense Federal University, Rio de Janeiro 24220-900, Brazil; 7 Department of Endodontics, Rio de Janeiro University (UERJ), Rio de Janeiro 20550-013, Brazil; 8 Department of Clinical Sciences, College of Dentistry, Ajman University, Ajman P.O. Box 346, United Arab Emirates; 9 CENIMAT/I3N, Department of Materials Science, NOVA School of Science and Technology, Universidade NOVA de Lisboa, 2829-516 Caparica, Portugal; 10 Dental Specialty Center, Brazilian Military Police, Belo Horizonte 30350-190, Brazil

**Keywords:** bending load, differential scanning calorimetry, endodontics, nickel-titanium alloy, root canal treatment, torsional strength

## Abstract

The present research compared the design, metallurgical properties, and mechanical characteristics of the ProTaper Ultimate instruments with five multifile systems. A total of 469 new nickel–titanium rotary finishing instruments, all 25 mm in length but varying in size, taper, and metal alloy composition, from six different multifile systems (ProTaper Ultimate, ProTaper Next, ProFile, Mtwo, EndoSequence, and GT Series X), were inspected for irregularities and analyzed for their spiral density (spirals per millimetre), blade design, surface finishing, alloy composition, phase transformation temperatures, and mechanical performance (microhardness, torsional, and bending resistance tests). Group comparisons were performed using Kruskal–Wallis and one-way ANOVA with post hoc Tukey’s tests (α = 5%). ProFile instruments exhibited a greater number of spirals and a higher density of spirals per millimetre compared to the other systems. Microscopic analysis revealed distinct tip geometries and blade designs among tested instruments. All of them displayed parallel marks from the machining process, but the EndoSequence system had the smoothest surface finish. The alloys of all instruments consisted of an almost equiatomic ratio of nickel to titanium. At the testing temperature, the ProTaper Ultimate system exhibited a complete R-phase crystallographic arrangement, while the ProFile and Mtwo systems were fully austenitic. The ProTaper Ultimate F2, F3, and FX instruments demonstrated the highest maximum torque values (1.40, 1.45, and 3.55 N.cm, respectively) and the lowest maximum bending loads (202.7, 254.9, and 408.4 gf, respectively). EndoSequence instruments showed the highest angles of rotation, while the highest microhardness values were recorded for GT Series X (407.1 HVN) and ProTaper Next (425.0 HVN) instruments. The ProTaper Ultimate system showed a high spiral density per millimetre and a complete R-phase crystallographic arrangement at room temperature, which significantly contributed to its superior flexibility and torsional strength when compared to the other tested systems.

## 1. Introduction

Root canal therapy aims to thoroughly clean and shape the root canal system while preserving the structural integrity of the teeth [1]. The development of nickel-titanium (NiTi) rotary systems has revolutionized root canal preparation, offering greater flexibility, efficiency, and safety compared to traditional stainless-steel instruments [1,2]. These benefits arise from the unique properties of NiTi alloys, which allow for better adaptation to complex canal anatomies and reduce the risk of procedural errors like ledging or transportation. However, although NiTi instruments have revolutionized root canal shaping, their susceptibility to deformation and fracture remains a key challenge [3]. To address this, manufacturers have focused on improving the durability and performance of these instruments by refining their design and developing thermally treated NiTi alloys. These advancements enhance flexibility and resistance to torsional failure, ultimately improving the safety and reliability of root canal treatment [2,4].

Since the launch of the first commercial NiTi rotary instruments in 1991 [5], there have been numerous advancements and variations in their design and metallurgical properties. These developments have focused on enhancing performance, flexibility, and durability, reflecting the evolving needs of dental practitioners and the complexity of root canal systems. The ProFile instruments (Dentsply Maillefer, Ballaigues, Switzerland) were among the first NiTi systems introduced to the market. These instruments are designed with three evenly spaced U-shaped grooves, a bullet-nosed tip with a rounded transition angle, and symmetrical radial lands aimed to decrease the risk of canal transportation and procedural errors [6]. While this system is well-regarded for its safety and reliable performance, its design inherently results in reduced cutting efficiency and higher torque requirements which can limit its effectiveness in specific clinical scenarios [1,7]. The GT Series X (Dentsply Maillefer) is a heat-treated, taper-centric multifile system with a radial-landed cross-section design that incorporates larger taper sizes while maintaining a limited maximum flute diameter and a rounded, passive tip, thereby enhancing both efficiency and safety during root canal shaping [8]. The EndoSequence system (Brasseler USA; Savannah, GA, USA) is made of conventional NiTi alloy and features a triangular cross-section with alternating contact points along the cutting length to enhance flexibility, electropolishing for increased durability, and variable pitch and helical angles that minimize the risk of the instrument screwing into the canal [9]. The ProTaper Next system (Dentsply Maillefer), widely recognized as one of the most popular root canal instrumentation systems, features a variable taper design and an off-centred rectangular cross-section, and is made using M-Wire technology to improve resistance to both cyclic fatigue and torsional stress [10,11]. The MTwo system (VDW, Munich, Germany), designed with an S-shaped cross-section featuring two cutting edges, a positive rake angle, and an increasing pitch length, is consistently reported to prepare root canals more quickly while effectively maintaining canal curvature [12,13]. All of these systems have been established in clinical practice for many years and are regarded as reliable options, demonstrating a proven track record of safety and efficiency in endodontic treatment. Their widespread acceptance among dental professionals ensures their continued use globally, even as newer systems are developed and introduced into the market [14,15,16,17].

In 2022, the ProTaper Ultimate (Dentsply Sirona Endodontics, Ballaigues, Switzerland) multifile system, the latest generation of ProTaper instruments, was launched. This system incorporates several novel features, including a parallelogram-shaped cross-section, specific heat-treated alloys for different instruments (M-wire, Gold, and Blue), and a limited maximum flute diameter of 1 mm (except for SX and FX instruments) [18]. A recent publication has compared the novel ProTaper Ultimate system to the ProTaper Universal (Dentsply Maillefer) and ProTaper Gold (Dentsply Sirona Endodontics) systems, concluding that ProTaper Ultimate exhibits lower torsional strength but superior flexibility [19]. While comparing this system with other instruments within the ProTaper family is essential for understanding their evolution, it is also important to assess its performance against well-established systems that have a proven track record in laboratorial studies and clinical practice. Such comparisons will enhance our understanding of the potential advantages of the ProTaper Ultimate in various clinical scenarios, providing evidence-based guidance for clinicians in selecting the most suitable system for specific situations. Furthermore, the currently available information on the mechanical behaviour of the ProTaper Ultimate system remains limited [19,20,21,22], highlighting the need for further investigation.

This study aims to provide a comprehensive comparative analysis of the ProTaper Ultimate finishing instruments alongside five other established and widely used NiTi rotary multifile systems—EndoSequence, ProFile, GT Series X, ProTaper Next, and MTwo—all of which feature distinct alloys and geometries, including the analysis of their design, metallurgical properties, and mechanical characteristics. The null hypothesis to be tested is that there is no significant difference in the mechanical performance between the new instruments of the ProTaper Ultimate system and the other tested systems.

## 2. Materials and Methods

### 2.1. Samples Selection

A total of 469 new NiTi rotary finishing instruments, 25 mm in length but varying in size, taper, and metal alloy composition, from 6 different multifile systems (ProTaper Ultimate, ProTaper Next, ProFile, Mtwo, EndoSequence, and GT Series X) were tested (Table 1). Each instrument was examined under a dental operating microscope (×13.6) (Opmi Pico, Carl Zeiss Surgical, Jena, Germany) for any major defects that could lead to exclusion from the study; none were excluded.

### 2.2. Design Assessment

Six instruments from each size were randomly selected and examined under a dental microscope (Opmi Pico) at ×13.6 magnification, with images captured using a digital camera (Canon EOS 500D; Canon, Tokyo, Japan) to evaluate the length of the active blade, number of spirals, spirals per millimetre, helical angle (calculated as the average of the six most coronal angles on the active blade), and spiral direction. The instruments were then mounted on a file holder and analyzed using a scanning electron microscope (SEM) (S-2400, Hitachi, Tokyo, Japan) to assess spiral geometry (symmetrical or asymmetrical), tip design (active or non-active), presence and types of surface marks from the machining process, and any minor manufacturing defects.

### 2.3. Metallurgical Characteristics

Metallurgical analysis was performed on reference instruments from each multifile system, including ProFile 30/.06, EndoSequence 30/.06, GT Series X 30/.06, ProTaper Next X3, MTwo 30/.06, and ProTaper Ultimate F3 and FX. Only one reference instrument was selected from each system, as all within a given system undergo the same metallurgical treatment. However, for the ProTaper Ultimate system, both the F3 and FX instruments were analyzed due to their distinct metallurgical treatments.

A semi-quantitative elemental analysis was accomplished on 3 reference instruments from each system using energy-dispersive X-ray spectroscopy (EDS) with a conventional SEM unit (DSM-962, Carl Zeiss Microscopy GmbH, Jena, Germany) [4,22] equipped with an Inca X-act EDS detector (Oxford Instruments NanoAnalysis, Abingdon, United Kingdom). The SEM was operated at 20 kV and 3.1 amperes, with an initial vacuum process of 10 min. Data collection was performed over a 500 µm × 400 µm area for 1 min at a working distance of 25 mm. The ZAF correction method was applied, and the proportions of metallic elements were calculated using specialized software (Microanalysis Suite v.4.14; Oxford Instruments NanoAnalysis, Abingdon, UK).

Differential scanning calorimetry (DSC) tests (DSC 204 F1 Phoenix; Netzsch-Gerätebau GmbH, Selb, Germany) were carried out to determine phase transformation temperatures, following ASTM F2004-17 [23] guidelines and previous publications [4,22]. A 4 to 5 mm fragment (weighing 5 to 10 mg) was cut from the active blade of each reference instrument and immersed in an etching bath (45% nitric acid, 25% hydrofluoric acid, and 30% distilled water) for 2 min. After the acid was neutralized with distilled water, each specimen was placed in an aluminum pan inside the DSC device, with an empty pan serving as the control. The thermal cycle, lasting 1 h 40 min, was performed under nitrogen gas protection. The temperature range for the cycle was set from −150 °C to 150 °C, increasing at a rate of 10 °C per minute. DSC data and graphs were created using Netzsch Proteus Thermal Analysis software (v.7.1; Netzsch-Gerätebau GmbH, Selb, Germany).

### 2.4. Mechanical Performance

The mechanical performance of the instruments was assessed by evaluating their torsional and bending resistance according to international standards [24], along with their microhardness. Sample size calculations were based on the largest differences observed in the results of two assessed instruments after five initial measurements. For the torsion and bending tests, the results from the eight instruments with a tip size of 30 were used as the reference. For the microhardness tests, the same reference instruments from the metallurgical characteristics assessment were employed. Assuming an alpha error of 0.05 and a power of 80%, the calculated sample sizes for maximum torque (effect size: 0.70 ± 0.41; EndoSequence 30/.04 vs. ProTaper Next X3), angle of rotation (effect size: 349.00 ± 183.68; EndoSequence 30/.06 vs. ProTaper Next X3), maximum bending load (effect size: 512.61 ± 257.39; EndoSequence 30/.04 vs. ProFile 30/.06), and microhardness (effect size: 89.00 ± 54.95; EndoSequence 30/.06 vs. ProTaper Next X3) were 6, 7, 6, and 8 instruments, respectively. Consequently, the final sample size for both the torsion and bending tests was established at 8 instruments for all groups, while 12 indentations (4 indentations on 3 different instruments per group) were used for the microhardness assessment.

In the torsional test, the instruments were positioned vertically on a torsiometer (TT100, Odeme Dental Research, Luzerna, Santa Catarina, Brazil) and secured at the apical 3 mm. They were then rotated at a constant speed of 2 rpm in a clockwise direction until they fractured [4,19,24]. The maximum torque experienced before breaking (in N.cm) and the angle of rotation (in degrees) were recorded using specialized software (Odeme Analysis TT100, Odeme Dental Research, Luzerna, Santa Catarina, Brazil). For the bending test, the instruments were fixed in a file holder at a 45° angle relative to the floor, with their apical 3 mm attached to a wire linked to a universal testing machine (DL-200 MF, EMIC, São José dos Pinhais, Brazil). A load of 20 N was applied at a constant speed of 15 mm/min until the instrument reached a 45° displacement [4,19,24]. The maximum load required to achieve this displacement was measured in gram-force (gf) using Tesc v3.04 software (Mattest Automação e Informática, Poá, Brazil). Microhardness testing was performed by creating indentations on the reference instruments using a Vickers hardness tester (Duramin; Struers Inc., Cleveland, OH, USA). Each specimen was prepared according to ASTM standards [25] and secured to an acrylic block before testing. Indentations were made with a diamond indenter under a load of 100 gf for 15 s [26], and the results were expressed as a Vickers Hardness Number (HVN) at a magnification of ×40.

### 2.5. Statistical Analysis

The parameters for maximum torque, angle of rotation, and maximum bending load exhibited a non-Gaussian distribution (Shapiro–Wilk test, *p* < 0.05). Consequently, these parameters were analyzed using the nonparametric Kruskal–Wallis test, with outcomes expressed as medians and interquartile ranges. In contrast, the microhardness results followed a Gaussian distribution (Shapiro–Wilk test, *p* > 0.05) and were analyzed using one-way ANOVA followed by a post hoc Tukey’s test, with results presented as means and standard deviations. The significance level was set at 0.05 (SPSS v.22 for Windows; IBM SPSS Statistics, Chicago, IL, USA).

## 3. Results

All ProFile instruments exhibited a greater number of spirals (n = 19), a higher density of spirals per millimetre (1.19 spirals/mm), and helical angles ranging from 40° to 50°, followed by the ProTaper Ultimate instruments, while the instruments with the fewest spirals were those from the EndoSequence and MTwo systems. All tested instruments had spirals oriented in a clockwise direction (Table 1).

**Figure 1:**
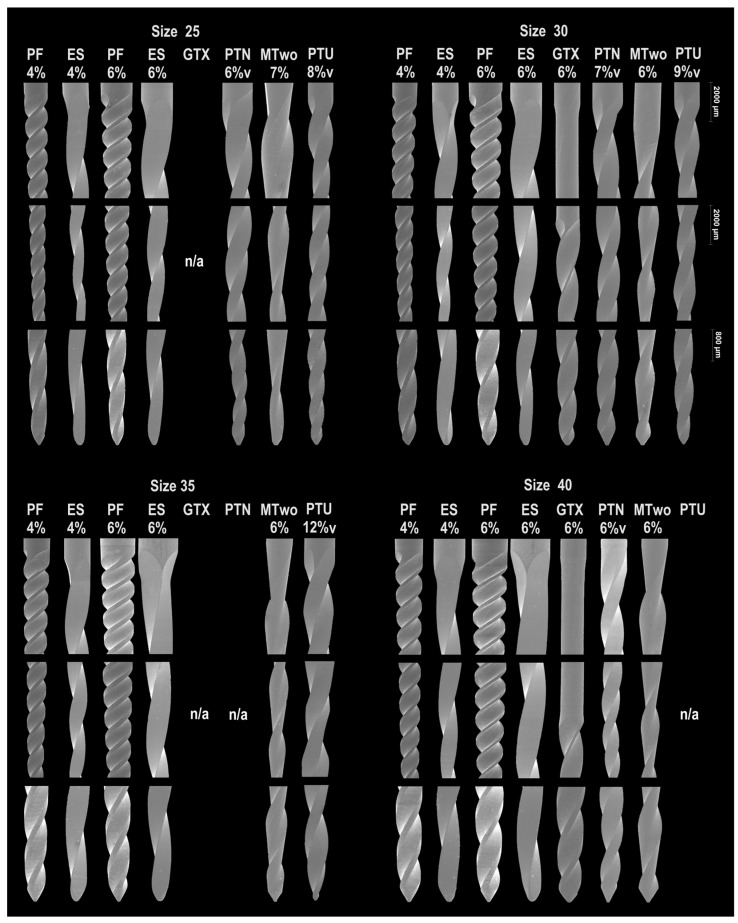
Representative SEM images show the coronal (magnification: 20×; HV: 20.0 kV), middle (magnification: 20×; HV: 20.0 kV), and apical (magnification: 40×; HV: 20.0 kV) portions of the 28 assessed instruments, categorized by tip size. All instruments displayed unique blade and tip designs. Areas marked as “n/a” indicate that a specific tip size is not available for that particular system.

SEM analysis revealed distinct tip geometries and blade designs among instruments, with none exhibiting an asymmetric design. Radial lands were present on both the ProFile and GT Series X instruments (Figure 1).

**Figure 2:**
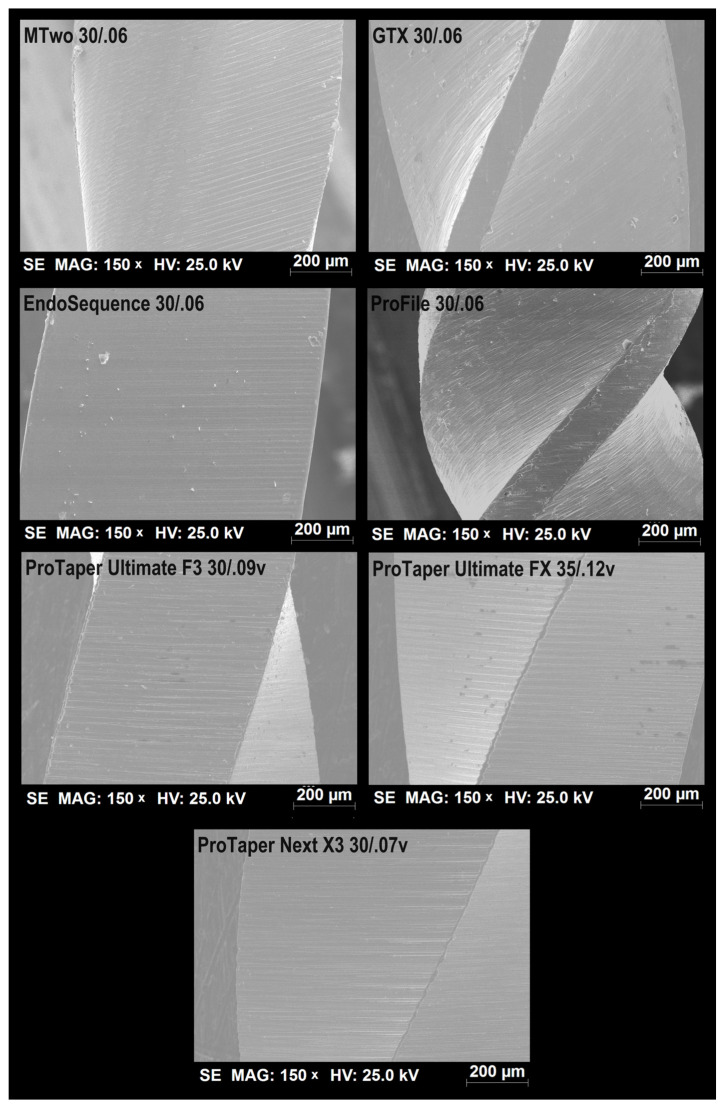
Representative SEM images showing the surface finish of the tested instruments reveal that all instruments exhibited characteristic parallel marks from the machining process. However, the EndoSequence system appeared to have the smoothest surface among them.

All tested instruments displayed parallel machining marks on their surfaces. However, the ProFile instruments had a more irregular surface due to these marks, while the EndoSequence instruments appeared smoother. In contrast, the surface finishes of the ProTaper Ultimate, ProTaper Next, and MTwo instruments were found to be comparable to each other (Figure 2).

The EDS test revealed that all reference instruments were composed of NiTi alloys with an almost equiatomic ratio of nickel and titanium, with no traces of other metal elements (Table 2).

**Figure 3:**
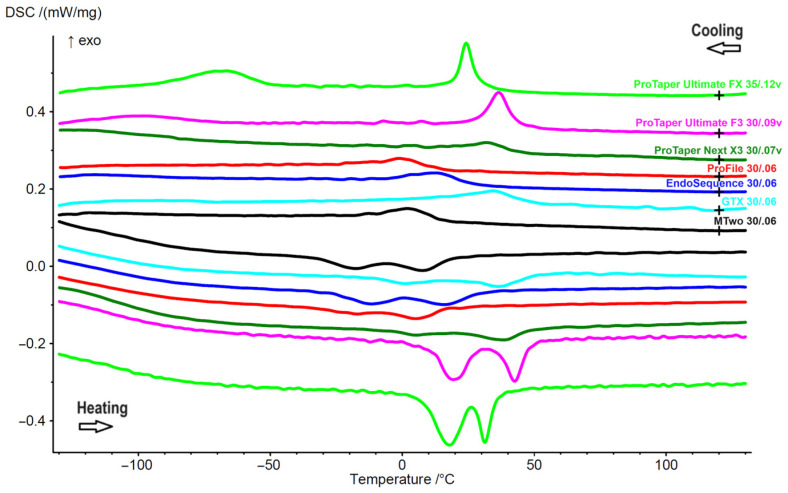
DSC chart illustrates the phase transformation temperatures of reference instruments for each rotary system. The upper lines represent the cooling curves (read from right to left), while the lower lines depict the heating curves (read from left to right). The data indicate that ProTaper Ultimate F3 and FX exhibit a complete R-phase crystallographic structure at room temperature, whereas ProFile and MTwo are fully austenitic. ProTaper Next and GT Series X show equivalent curves.

The DSC tests showed distinct phase transformation temperatures among the instruments. The ProTaper Ultimate F3 exhibited the highest R-phase start (Rs: 45.1 °C) and finish (Rf: 32.7 °C) temperatures, while the ProFile instruments had the lowest values (Rs: 15.2 °C and Rf: −26.8 °C). Notably, the ProTaper Ultimate instruments (F3 and FX) were the only ones to display a complete R-phase crystallographic arrangement at room temperature (20 °C). In contrast, the GT Series X, ProTaper Next, and EndoSequence instruments exhibited a mixed austenitic and R-phase structure, while both MTwo and ProFile were fully austenitic. As the temperature increased from room temperature (20 °C) to body temperature (36 °C), the ProTaper Ultimate F3, ProTaper Next, and GT Series X instruments exhibited a mixed austenitic and R-phase crystallographic arrangement, whereas all other instruments remained fully austenitic (Figure 3, Table 2).

**Figure 4:**
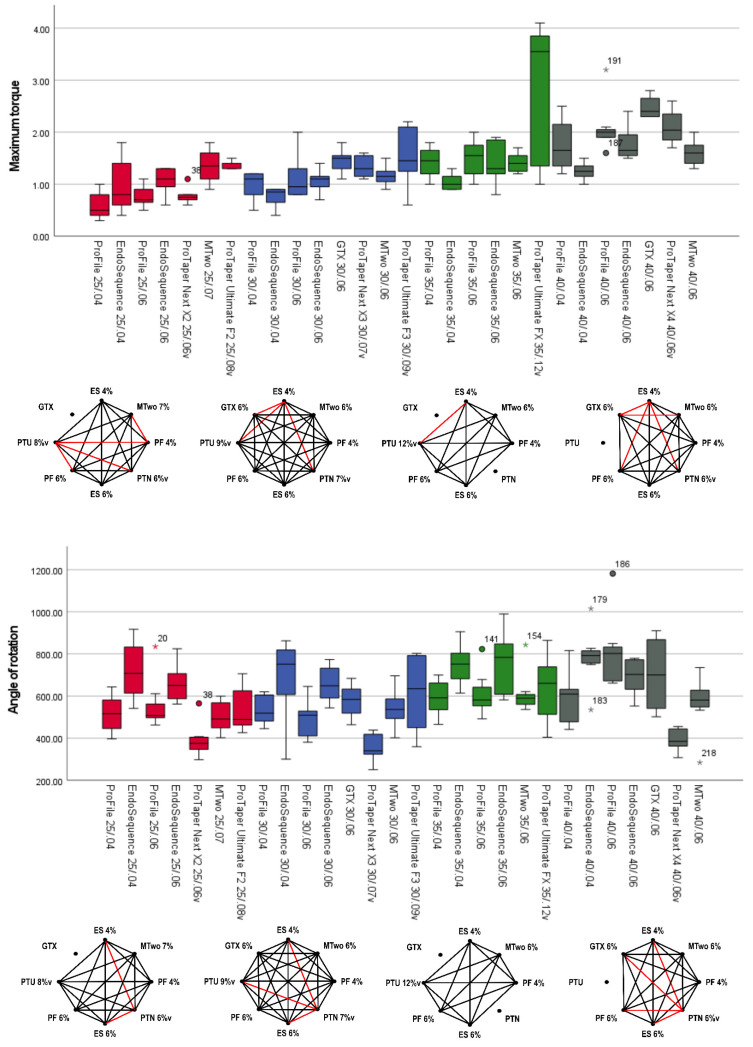
The boxplot charts display the maximum torque (**top**) and angle of rotation (**bottom**) results for the 28 assessed instruments. Each boxplot indicates the median (the line in the middle of the rectangle) and the interquartile range. The octagonal charts highlight statistically significant differences (*p* < 0.05) between instruments with the same tip size, as represented by the red lines. The ProTaper Ultimate generally exhibited higher maximum torque values, whereas both the 0.04 and 0.06 EndoSequence instruments demonstrated greater angles of rotation (dots and asterisks represent outliers).

In the torsional test, ProTaper Ultimate instruments generally exhibited higher maximum torque values compared to other instruments of the same tip size (*p* < 0.05). ProTaper Ultimate F2 had a median maximum torque value of 1.40 N.cm, while conventional NiTi instruments, such as ProFile and EndoSequence, showed values below 1.10 N.cm. The ProTaper Ultimate F3 reached 1.45 N.cm, a value only matched by the GT Series X 30/.06 (1.50 N.cm). The ProTaper Ultimate FX demonstrated a significantly higher maximum torque of 3.55 N.cm, compared to other instruments of the same tip size, which reached values below 1.55 N.cm, as well as instruments with a tip size of 40. Overall, larger apical sizes were associated with higher maximum torque values in the torsional test (Figure 4, Table 3). In terms of angle of rotation, the highest values were observed in the EndoSequence instruments, while the lowest were recorded in the ProTaper Next, regardless of tip size. The ProTaper Ultimate displayed well-balanced performance compared to other tested instruments within the same tip size group. Additionally, an increase in tip size did not significantly impact the angle of rotation results (Figure 4, Table 3).

**Figure 5:**
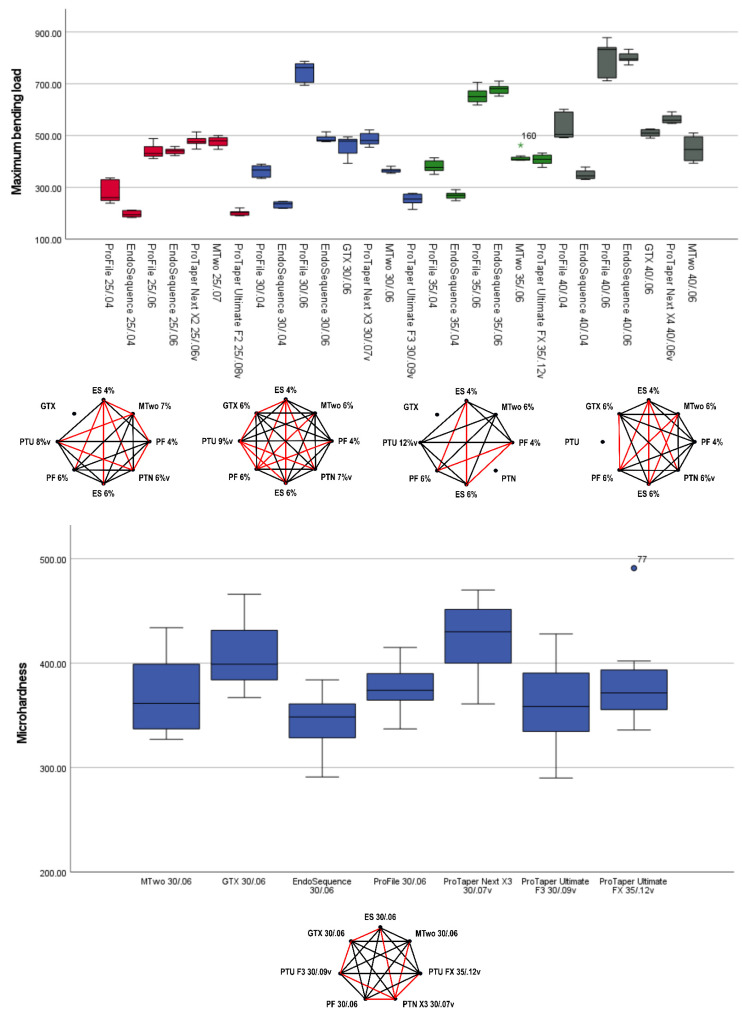
The boxplot charts present the results for maximum bending load (**top**) and microhardness (**bottom**) for all tested instruments. Each boxplot displays the median results along with the interquartile range, while the octagonal charts highlight statistically significant differences between instruments, indicated in red. The ProTaper Ultimate and 0.04 taper EndoSequence instruments demonstrated the lowest maximum bending load, suggesting greater flexibility. In contrast, the GT Series X and ProTaper Next instruments exhibited higher microhardness values (dots and asterisks represent outliers).

The lowest maximum bending loads (indicating higher flexibility) were observed in the EndoSequence 0.04 taper instruments (25/.04: 194.3 gf; 30/.04: 236.8 gf; 35/.04: 269.3 gf; 40/.04: 344.4 gf), which were comparable to ProTaper Ultimate instruments of similar tip sizes: F2 (202.7 gf), F3 (254.9 gf), and FX (408.4 gf) (*p* > 0.05). Instruments with larger tip sizes, along with ProFile and EndoSequence instruments with a 0.06 taper, tended to show higher maximum bending loads (indicating less flexibility). Except for the EndoSequence 40/.04, all instruments with a tip size of 40 and 0.04 or 0.06% tapers were less flexible than the ProTaper Ultimate FX (size 35, 0.12 taper) (Figure 5, Table 3).

In the microhardness test, significantly higher values were observed in the alloys of GT Series X (407.1 HVN) and ProTaper Next (425.0 HVN) compared to all other alloys (*p* < 0.05), including the alloys of the ProTaper Ultimate F3 (Gold wire) and FX (Blue wire), which showed no significant differences between them (*p* > 0.05) (Figure 5, Table 3).

## 4. Discussion

Clinical challenges, along with advancements in technology and manufacturing capabilities, drive the continuous development of new rotary preparation systems in the market. Although commercial brands provide detailed specifications for these systems, it is essential for independent research to verify these claims and determine whether the new features truly offer performance improvements that justify adopting them in clinical practice. In many cases, the innovations may not represent a significant enough advancement to warrant clinics updating or replacing their existing inventories. This study offers valuable insights into the mechanical and metallurgical properties of the new ProTaper Ultimate instruments, demonstrating their superior flexibility and torsional resistance in comparison to five other widely used multifile systems. Based on the observed differences among instruments in the same category, the null hypothesis was rejected.

In terms of maximum torque outcomes, larger tip sizes or larger tapers within the same tip size category tend to exhibit higher values, corroborating previous findings [27]. Although conventional NiTi alloys have been associated with higher maximum torque values compared to heat-treated alloys [28,29], the heat-treated ProTaper Ultimate system displayed higher maximum torque values than conventional NiTi systems such as ProFile, EndoSequence, and MTwo across most tip size categories (Figure 4, Table 3). This may be attributed to the larger dimensions of the ProTaper Ultimate instruments at D3 in each size group, as the ISO torsional test uses this specific location as the reference point for evaluation. This suggests superior resistance to torsional failure, a critical factor in preventing instrument breakage during clinical use [2,4]. Interestingly, these findings differ from previous research that reported higher torque values for conventional NiTi instruments from the ProTaper Universal system compared to the ProTaper Ultimate [19], likely due to the similar design and cross-sectional areas of both systems. However, the results align with studies comparing the F2 Ultimate instrument to other heat-treated systems such as RSC Rainbow (Ramo Medical, Suzhou, China), RaCe Evo (FKG, La Chaux-de-Fonds, Switzerland), Rotate (VDW, Munich, Germany), and One Curve N25 (Micro-Mega, Besançon, France) [22]. The ProTaper Ultimate system does not include an instrument with a tip size 40, but the other systems with this tip size were evaluated in the study. Notably, the ProTaper Ultimate F3 (tip size 30) exhibited maximum torque values comparable to conventional NiTi instruments with a tip size 40, while the ProTaper Ultimate FX (tip size 35) outperformed all other size 35 or 40 instruments (Figure 4, Table 3). This superior performance may again be attributed to the larger apical dimensions of ProTaper Ultimate instruments.

Regarding the angle of rotation, which reflects the instrument’s ability to resist deformation before fracturing under torsional stress, both the 0.04 and 0.06 taper EndoSequence instruments consistently exhibited higher rotation angles before fracture during torsional testing. While conventional NiTi alloys are generally associated with reduced flexibility and, consequently, lower rotation angles [4], the EndoSequence instruments seem to overcome this limitation. This may be attributed to their triangular cross-section, which likely reduces core mass, and an electropolished surface that smooths the instrument, potentially slowing crack propagation [30], thus increasing rotation angles before fracture. The ProTaper Ultimate instruments also demonstrated rotation angles comparable to those of EndoSequence (Figure 4, Table 3).

The maximum bending load, which reflects instrument flexibility, was another mechanical parameter evaluated in this study. The ProTaper Ultimate exhibited lower values (indicating greater flexibility), performing similarly to the 0.04 taper EndoSequence instruments. In contrast, higher maximum bending loads (indicating reduced flexibility) were observed in the 0.06 ProFile and EndoSequence, as well as in the MTwo and ProTaper Next instruments (Figure 5, Table 3). Despite having a larger tip size of 35, the ProTaper Ultimate FX showed low maximum bending loads, making it more flexible than several instruments with tip sizes 25, 30, or 40 from the 0.06 taper ProFile, EndoSequence, MTwo, GT Series X, and ProTaper Next instruments. Moreover, despite their greater apical taper, the ProTaper Ultimate instruments demonstrated a high angle of rotation and low maximum bending load, reflecting good flexibility. This suggests that this system effectively compensates for torque stresses and may minimize canal transportation, making it particularly suitable for navigating complex root canal anatomies, as supported by a recent shaping ability study [20]. Additionally, previous studies confirm the superior flexibility of ProTaper Ultimate, as demonstrated by both their angle of rotation and maximum bending load, when compared to other instruments, regardless of whether those instruments were made from conventional or heat-treated NiTi alloys [19,22]. Flexibility is key to reducing procedural errors and preserving the integrity of the root canal anatomy, especially in cases with severe curvature5. It also enhances resistance to cyclic fatigue, as shown in a recent report [21], where the ProTaper Ultimate F2 outperformed the ProTaper Gold F2 (Dentsply Sirona Endodontics) and 25/.08 M3 (United Dental Group, Changzhou, China) instruments.

The high flexibility of ProTaper Ultimate instruments can be attributed to several key characteristics, including a high number of spirals per millimetre—surpassed only by ProFile instruments—which is associated with enhanced flexibility [5]. Additionally, their heat treatment results in a complete R-phase crystallographic arrangement at the testing temperature (Figure 3, Table 2). This contrasts with the fully austenitic structure found in ProFile and MTwo instruments, as well as the mixed austenitic and R-phase structure of the other systems. Furthermore, the presence of a mixed austenitic and R-phase structure at body temperature in the ProTaper Ultimate system (Figure 3) suggests favourable mechanical properties, striking a balance between stiffness and flexibility at those temperatures. In comparison, the other systems typically achieve fully austenitic features at the same temperature, which provides them with higher torque values but reduced flexibility.

In the present study, both M-wire systems—GT Series X and ProTaper Next—demonstrated significantly higher microhardness values of 407.1 and 425.0 HVN, respectively, compared to all other systems, which ranged from 345.3 to 380.0 HVN (Figure 5, Table 2). These results are consistent with a previous study that found M-wire to be harder than conventional NiTi wire [31]. This suggests that M-wire instruments possess greater hardness, potentially improving their resistance to wear and deformation [31] compared to similar instruments made of other alloy types.

The strengths of the present study include its multimethod approach, which provides a more comprehensive understanding of the tested instruments [32] and the use of multiple instruments from the same systems. This allows for a more thorough evaluation of each system as a whole rather than focusing on a single instrument. Furthermore, the comparison of five distinct systems made from two different alloys (M-Wire and conventional NiTi) and featuring five different geometries offers a robust basis for comparison with other preparation systems available on the market. However, the study has limitations, including the absence of other relevant tests, such as cyclic fatigue assessments or evaluations of cutting ability, or more clinically oriented methods such as micro-CT shaping ability assessment in natural teeth [20,33,34], as well as the lack of comparison with additional heat-treated NiTi systems, or more metallurgical assessments. Therefore, further research on ProTaper Ultimate instruments should incorporate the evaluation of additional systems and more comprehensive testing including more clinical oriented approaches.

## 5. Conclusions

This study compares the ProTaper Ultimate with five other NiTi multifile systems, highlighting differences in design, mechanical properties, and metallurgy. ProTaper Ultimate stood out for its high spiral density and full R-phase crystallographic structure, enhancing flexibility and torsional strength. ProFile had the highest spiral density, while EndoSequence showed the smoothest surface. The lowest torque values were observed in ProFile (0.04 taper) and EndoSequence, the latter being the most flexible. Instruments made of M-wire alloy, such as GT Series X and ProTaper Next, had higher microhardness, improving wear resistance. MTwo and ProFile were fully austenitic. All systems had a near to equiatomic NiTi ratio. The study emphasizes the role of design and materials in NiTi rotary system performance, offering insights for clinicians in choosing the best instruments for root canal treatment.

## Figures and Tables

**Table 1:** Geometric design characteristics of 28 NiTi rotary instruments from 6 different systems.

Instruments	Apical Size/Taper	Active Blade Length (mm)	Number of Spirals	Spirals per Millimetre	Helical Angle (°)	Lot Number
	Tip size 25					
ProFile 25/.04	25/.04	16	19	1.19	40.1 ± 2.4	6519600
EndoSequence 25/.04	25/.04	16	8	0.50	14.7 ± 2.8	0198
ProFile 25/.06	25/.06	16	19	1.19	48.6 ± 1.6	1720487
EndoSequence 25/.06	25/.06	16	7	0.44	13.7 ± 2.8	9376
ProTaper Next X2	25/.06v	17	8	0.47	20.8 ± 2.5	1515010
MTwo 25/.07	25/.07	17	6	0.35	18.7 ± 4.5	363436
ProTaper Ultimate F2	25/.08v	17	16	0.94	18.9 ± 1.2	1854170
	Tip size 30					
ProFile 30/.04	30/.04	16	19	1.19	42.7 ± 2.0	2725220
EndoSequence 30/.04	30/.04	16	8	0.50	14.7 ± 1.8	0198
ProFile 30/.06	30/.06	16	19	1.19	47.3 ± 1.4	060308511
EndoSequence 30/.06	30/.06	16	7	0.44	14.8 ± 1.0	9376
GT Series X 30/.06	30/.06	12	8	0.67	23.0 ± 4.5	SXRAS25
ProTaper Next X3	30/.07v	17	7	0.41	22.3 ± 2.7	1515010
MTwo 30/.06	30/.06	17	7	0.41	20.9 ± 1.7	362601
ProTaper Ultimate F3	30/.09v	17	12	0.71	18.6 ± 1.5	1824935
	Tip size 35					
ProFile 35/.04	35/.04	16	19	1.19	42.0 ± 2.1	7235110
EndoSequence 35/.04	35/.04	16	7	0.44	15.8 ± 2.3	0198
ProFile 35/.06	35/.06	16	19	1.19	48.0 ± 2.0	1712662
EndoSequence 35/.06	35/.06	16	6	0.38	13.9 ± 4.0	9376
MTwo 35/.06	35/.06	17	8	0.47	21.2 ± 1.1	0903310641
ProTaper Ultimate FX	35/.12v	17	8	0.47	19.1 ± 2.0	1824935
	Tip size 40					
ProFile 40/.04	40/.04	16	19	1.19	43.7 ± 2.7	7051790
EndoSequence 40/.04	40/.04	17	6	0.35	11.9 ± 1.7	0198
ProFile 40/.06	40/.06	16	19	1.19	50.6 ± 2.6	1686802
EndoSequence 40/.06	40/.06	17	5	0.29	11.9 ± 3.0	9376
GT Series X 40/.06	40/.06	10	7	0.70	22.3 ± 1.5	SXRAS25
ProTaper Next X4	40/.06v	18	7	0.39	19.1 ± 3.2	1529960
MTwo 40/.06	40/.06	17	8	0.47	24.5 ± 1.6	0904310642

**Table 2:** Phase transformation temperatures (°C), elemental composition (%), and the microhardness (HVN) of the tested reference instruments made from different NiTi alloys from each system.

Instruments	NiTi Wire	Phase Transformation Temperatures				Elemental Composition			Microhardness
		Rs	Rf	As	Af	Nickel	Titanium	Ni/Ti Ratio	
MTwo 30/.06	Conventional	15.8	−12.2	−32.6	19.6	51.42	48.58	1.058	370.0 ± 36.9
GT Series X 30/.06	M-wire	50.2	11.8	−9.9	50.3	50.23	49.77	1.009	407.1 ± 30.7
EndoSequence 30/.06	Conventional	27.9	−4.5	−28.8	30.4	51.50	48.50	1.062	345.3 ± 26.1
ProFile 30/.06	Conventional	15.2	−26.8	−33.2	17.0	51.78	48.22	1.074	376.5 ± 19.9
ProTaper Next X3	M-wire	45.3	16.3	−3.1	49.6	50.32	49.68	1.013	425.0 ± 32.9
ProTaper Ultimate F3	Gold	45.1	32.7	9.1	51.1	51.08	48.92	1.044	361.7 ± 39.7
ProTaper Ultimate FX	Blue	32.6	21.1	5.6	34.6	50.55	49.45	1.022	380.0 ± 40.7

Rs: R-phase start, Rf: R-phase finish, As: austenitic start, Af: austenitic finish.

**Table 3:** Median [interquartile range] for maximum torque, angle of rotation, and maximum bending load of 28 NiTi rotary instruments from 6 different systems.

Instruments	Torsional Test		Bending Test
	Maximum Torque (N.cm)	Angle of Rotation (°)	Maximum Bending Load (gf)
Tip size 25			
ProFile 25/.04	0.50 [0.40–0.80]	516.7 [430.1–592.8]	260.9 [246.3–330.5]
EndoSequence 25/.04	0.80 [0.60–1.50]	707.4 [601.6–850.6]	194.3 [186.4–210.9]
ProFile 25/.06	0.70 [0.63–0.90]	508.6 [495.7–586.5]	430.7 [417.3–465.3]
EndoSequence 25/.06	1.10 [0.93–1.30]	649.5 [580.5–717.4]	440.9 [429.6–448.3]
ProTaper Next X2 25/.06v	0.75 [0.70–0.80]	375.3 [338.2–406.2]	475.9 [467.7–491.8]
MTwo 25/.07	1.35 [1.10–1.60]	490.9 [447.2–570.9]	479.6 [461.4–495.6]
ProTaper Ultimate F2 25/.08v	1.40 [1.30–1.40]	489.1 [460.3–630.1]	202.7 [192.4–206.9]
Tip size 30			
ProFile 30/.04	1.10 [0.75–1.20]	518.7 [474.3–609.3]	367.0 [338.1–386.5]
EndoSequence 30/.04	0.85 [0.58–0.90]	750.8 [606.1–836.8]	236.8 [220.2–244.6]
ProFile 30/.06	0.95 [0.80–1.35]	509.4 [409.9–530.3]	762.2 [701.2–778.2]
EndoSequence 30/.06	1.10 [0.93–1.18]	648.4 [579.5–735.3]	481.8 [477.7–495.4]
GT Series X 30/.06	1.50 [1.25–1.58]	584.7 [495.9–644.1]	478.6 [414.9–486.9]
ProTaper Next X3 30/.07v	1.30 [1.13–1.58]	339.9 [321.7–419.6]	480.9 [465.7–511.2]
MTwo 30/.06	1.15 [1.03–1.28]	536.8 [478.4–591.7]	366.4 [358.2–369.9]
ProTaper Ultimate F3 30/.09v	1.45 [1.23–2.15]	634.2 [408.3–791.7]	254.9 [239.2–275.2]
Tip size 35			
ProFile 35/.04	1.45 [1.15–1.68]	591.6 [523.5–673.2]	376.9 [364.9–403.7]
EndoSequence 35/.04	1.00 [0.90–1.18]	751.2 [661.1–811.8]	269.3 [255.6–278.9]
ProFile 35/.06	1.55 [1.20–1.78]	581.9 [551.4–659.4]	650.3 [624.4–677.7]
EndoSequence 35/.06	1.30 [1.15–1.88]	782.9 [597.3–860.1]	681.3 [661.6–691.6]
MTwo 35/.06	1.40 [1.23–1.58]	590.3 [558.2–613.5]	406.8 [405.4–418.9]
ProTaper Ultimate FX 35/.12v	3.55 [1.33–3.87]	659.9 [504.5–743.6]	408.4 [387.9–426.6]
Tip size 40			
ProFile 40/.04	1.65 [1.28–2.23]	608.9 [464.7–633.8]	504.5 [493.7–591.6]
EndoSequence 40/.04	1.25 [1.13–1.38]	791.6 [752.3–820.5]	344.4 [333.8–367.6]
ProFile 40/.06	2.00 [1.90–2.08]	801.9 [666.3–840.7]	832.4 [720.4–840.7]
EndoSequence 40/.06	1.65 [1.53–2.08]	702.0 [629.6–773.4]	796.0 [789.7–823.5]
GT Series X 40/.06	2.40 [2.30–2.73]	699.4 [538.3–873.1]	509.7 [496.9–523.5]
ProTaper Next X4 40/.06v	2.05 [1.83–2.43]	385.1 [358.7–445.8]	559.5 [548.4–580.1]
MTwo 40/.06	1.60 [1.40–1.78]	580.9 [541.1–639.5]	446.2 [402.4–497.8]

## Data Availability

The original contributions presented in this study are included in the article. Further inquiries can be directed to the corresponding author.
